# A Few Notes on Dental Prosthetics

**Published:** 1896-03

**Authors:** S. Globensky

**Affiliations:** Montreal.


ARTICLE III.

### A FEW NOTES ON DENTAL PROSTHETICS.

BY S. GLOBENSKY, L. D. S., MONTREAL.

Although we must regard the necessity for artificial dentures as great a reproach to dental practice as the necessity for surgery is to the general practitioner, yet we are forced to admit that prosthetics and surgery are indispensable, and that it is our duty to raise them to the highest possible perfection.

There are two sides to the question as to whether or not the introduction of vulcanite has been a curse to our profession. I am disposed to believe that students are made now in more haste than when artificial substitutes were confined to the more difficult bases of gold and platinum; and I am also disposed to believe that there is a much larger percentage of rude and unskilful work put into the mouths of patients. It is a bad sign of the times when cheapness is the chief object of attainment. We know that in all other branches of industrial art mere cheapness is followed by inferior production. Yet it must be recognized, on the other hand, that vulcanite has brought artificial substitutes more within the reach of the middling and the poorer classes; and yet, for this very reason, I am disposed to believe that it has made them more indifferent to the preservation of the natural teeth.

However, we cannot escape the fact that the public demand this class of dentistry, and it occurs to me that I may be permitted to suggest a few points mainly directed to the treatment of the mouth before inserting sets.

First of all, I would ask your attention to those cases where the alveolus has undue prominence, sometimes so as to cause positive disfiguration, protrusion of the lips and excessive projection of the teeth. It is a well-known fact that after extraction and the usual absorption of the alveolus, extending over a year, there remains an ugly exhibition of structure, to which it is sometimes impossible, and at all times difficult, to adapt an artistic set that will conceal its own art. Especially is this true, whether we use plain or gum teeth. If it is impossible to use the latter, or elevate the lip under the nostril by pink vulcanite—which, of course, turns dark and is a poor substitute for porcelain gum—then the depressions under and on each side of the nostrils, due to the removal of the long roots of the cuspids, make a permanent deformity. What shall we do in these cases?

1. As a rule, the cuspid roots are perhaps the most solidly fixed in the maxillary. As a rule, they can be easier treated for their preservation, because nature seems to resent their removal. I see no reason why it would not be wiser to excise than to extract their crowns, treat and fill them, and insert the set over the roots. As a rule, I think that the need for gum-teeth would not then be so urgent. Of course, to every rule there are exceptions. In case of such treatment, the patient should be instructed to have the roots examined once a year, for fear of elongation and bearing too much upon and weakening the plate.

2. There are very prominent protrusions which would be better treated surgically. After the teeth are extracted, and the gum-line and alveolus border still hang below the lip, there is no reason why the former may not be dissected from the alveolus, and the latter excised or cut down by forceps. In fact, the prospect of any undue protrusion after entire absorption can be effectually prevented by this precautionary surgery, and no possible injury inflicted upon the patient.

You will observe that I have not introduced any reference to the opportunities for crown and bridge work, as my object is to deal exclusively with the average demands upon the average practitioner and the means of the average patient.

3. It is not by any means a novel treatment to take an impression for a number of teeth before their extraction, cut them off the plaster model, set them up, and have them ready for insertion the moment they are extracted. But this has been largely limited to partial dentures. There is no reason why it may not be extended successfully for entire sets, if a good impression is secured, and the plaster teeth carefully cut from the model. It enables you also to replace the teeth, one by one, on the model, precisely in the position where nature puts them, and, after some experience, it will be easier to do this than to work upon the ragged plaster impression of wounded gaps, left after the forceps have been used. It will, too, be observed, that this is an advantage in the case of the extra-prominent mouths of which I have spoken. It is a fact that a set made in such a way may be inserted a few minutes after the teeth are all extracted, and worn without alteration for four or five years.

4. I attribute most fractures of the alveolus to hasty and rough attempts to extract. Of course, there are cases of distortion of roots, of exostosis, of osseous union with the socket, which makes the exception. But it is not difficult to learn, by previous examination, the condition of solidity of the teeth in the maxillary. In cases where this is very marked, it will follow that if the cuspids have to be extracted they will likely be the most difficult. Now, in order to avoid fractures, either of the teeth or of the processes, it is a wise precaution, if an anæsthetic can be prolonged, to begin at the cuspids and rapidly follow to the others, especially the first and second molars, and firmly twist or shake these teeth. You see, the object is to loosen the pericementum, which instaneously inflames, and within a minute the firmest tooth will much earier yield to the applied force of absolute extraction. It is a wrong principle to persist in tugging at an extremely difficult and solid tooth, risking its fracture and that of the surrounding alveolus. It is wiser to be slow and sure.

My attention has been drawn to these facts by observing a good deal of butchery in the human mouth, caused by ignorance and want of skill. Students get the idea that any one can extract a tooth, and, for some curious reason, anyone seems at liberty in the United States and Canada to do so, whether they do it with a piece of cord or a pair of tongs, and no matter whether they break the tooth, the alveolus, or the jaw! The result is that scientific surgery in this branch is rare, and as Heaven seems to take care of the fools who do not know enough to take care of themselves, much of this dental butchery has no serious result. On the other hand, the mouth is often disfigured for life. We find patients, who have been in those abbatoirs, with splintered sockets, torn gums and lips or tongue, inflamed mucous membrane, and. instead of a uniformly healed ridge, one of depressions and deformity. For this reason, I do not favor the use, in cases where extraction is seen to be difficult and prolonged, of anæsthetics which oblige us to hurry the operation.

I regret, Mr. Chairman and gentlemen, that I have been obliged to limit my remarks to these few pages, as I feel sure that they must touch upon the daily observation of each one of you, as well as of myself.—*Dominion Dental Jonrnal.*

